# Development of a Quantitative Detection System for *Porphyromonas gingivalis* Based on Immunochromatography

**DOI:** 10.3390/dj14070422

**Published:** 2026-07-09

**Authors:** Kaoru Kobayashi, Ryota Yamasaki, Michihiko Usui, Masahiro Morita, Maki Inoue, Yoshie Nagai-Yoshioka, Masanori Iwasaki, Keisuke Nakashima, Tatsuji Nisihara, Wataru Ariyoshi

**Affiliations:** 1 Division of Infections and Molecular Biology, Department of Advanced Pathophysiological Science, School of Dentistry, Kyushu Dental University, Kitakyushu 803-8580, Japan; r20kobayashi@fa.kyu-dent.ac.jp (K.K.); r18yamasaki@fa.kyu-dent.ac.jp (R.Y.); r21morita2@fa.kyu-dent.ac.jp (M.M.); r16yoshioka@fa.kyu-dent.ac.jp (Y.N.-Y.); tatsujin@kyu-dent.ac.jp (T.N.); 2 Oral Medicine Innovation Center, Kyushu Dental University, Kitakyushu 803-8580, Japan; 3 Division of Periodontology, Department of Oral Function, School of Dentistry, Kyushu Dental University, Kitakyushu 803-8580, Japan; r12usui@fa.kyu-dent.ac.jp (M.U.); nakashimak@kyu-dent.ac.jp (K.N.); 4 Division of Periodontology, School of Dentistry, Ohu University, Koriyama 963-8041, Japan; 5 Periodontal Medicine Center, Kitakyushu 806-0021, Japan; r20inoue@fa.kyu-dent.ac.jp; 6 Division of Dental Public Health, Department of Oral Health Science, Graduate School of Dental Medicine, Hokkaido University, Sapporo 060-8586, Japan; iwasaki@den.hokudai.ac.jp

**Keywords:** periodontitis, *Porphyromonas gingivalis*, immunochromatography

## Abstract

**Background**: *Porphyromonas gingivalis* is the principal bacterial species implicated in the pathogenesis of periodontitis. Despite its central role, direct detection of periodontopathic bacteria is not routinely performed in dental clinics. Although test kits using monoclonal antibodies against *P. gingivalis* have been reported, no practical chairside method has yet been established. **Objectives**: In this study, we developed an immunochromatography system that enables rapid and quantitative detection of *P. gingivalis* within 10 min. **Methods**: We evaluated the performance of the developed immunochromatography system and its dedicated measurement reader. **Results**: The kit demonstrated a minimum detection limit of 2.6 × 10^4^ CFU/mL and maintained detection capability across fimbrial types I and II. The dedicated reader showed strong correlations with a reference reader (*R*^2^ = 0.9921) and visual color chart evaluation (*R*^2^ = 0.9347). High intra- and inter-device precision was observed, with coefficients of variation less than 0.6%. Although the presence of 2% whole blood interfered with visual interpretation, other oral substances, including toothpastes and mouthwashes, did not significantly affect the assay performance. **Conclusions**: Collectively, these findings indicate that the developed system may serve as a rapid and reliable chairside tool for quantitative detection of *P. gingivalis*, with potential utility in the diagnosis and management of periodontitis and related systemic conditions.

## 1. Introduction

Periodontal diseases, excluding non-plaque-induced gingival diseases, are chronic infectious conditions characterized by inflammatory destruction of the alveolar bone and connective tissues caused by periodontopathic bacteria [1]. Poor oral hygiene conditions aid the adhesion of bacteria, primarily *Streptococcus* species, which adhere to the tooth surfaces, leading to dental plaque formation [2]. Progressive accumulation of dental plaque promotes the production of extracellular polysaccharides by co-aggregating bacteria, leading to the formation of biofilms. Dental plaque initially induces gingivitis, which can extend to the alveolar bone and progress to periodontitis.

The estimated prevalence of periodontitis in Japan is 49.4% [3]. It is a major cause of tooth loss in adults [4], resulting in impaired oral function and reduced quality of life [5,6]. Among the numerous bacterial species implicated in the pathogenesis of periodontitis, three species collectively known as the Red Complex, *Porphyromonas gingivalis*, *Treponema denticola*, and *Tannerella forsythia*, are strongly associated with periodontitis, as well as with various systemic diseases, including cardiovascular disease [7], diabetes [8] and Alzheimer’s disease [9].

In particular, *P. gingivalis* has been suggested to not only possess various virulence factors—such as lipopolysaccharide (LPS), fimbriae, and gingipain [10]—but also disrupt the oral immune homeostasis essential for maintaining oral health, thereby impairing host immune functions and promoting dysbiosis [11,12]. Therefore, the detection and monitoring of *P. gingivalis* are clinically crucial for determining periodontal treatment strategies and preventing the progression of periodontitis. In fact, *P. gingivalis* is frequently detected in subgingival plaque [13] and gingival crevicular fluid (GCF) [14] from patients with chronic periodontitis.

Current periodontal examinations in dental clinics and large-scale epidemiological studies primarily assess clinical parameters, including periodontal pocket depth, gingival index, and attachment level [15]; however, direct detection of periodontopathic bacteria is not routinely performed. Gene amplification-based methods such as polymerase chain reaction (PCR) [13,16] and isothermal amplification and lateral flow strip methods [14] have been developed and applied for bacteriological testing [16]. However, these tests are expensive for routine clinical screening. Although the development of test kits using monoclonal antibodies against *P. gingivalis* has been described, no practical chairside method has yet been reported. The development of a rapid chairside detection system for periodontopathic bacteria could facilitate more accurate diagnosis of periodontitis and enable the appropriate use of antibiotics, which would be of considerable clinical importance, particularly in the context of periodontitis-systemic disease interactions.

Immunochromatography (IC) tests are techniques that detect target analyses through specific antigen–antibody binding as the sample migrates along a membrane [17]. These tests offer several advantages, including operational simplicity, rapid assay time, long-term stability, and low cost [18]. Accordingly, IC techniques have been widely applied in the diagnosis of infectious diseases, such as influenza virus infection [19] and *Mycoplasma pneumoniae* infection [17].

In this study, we aimed to develop a rapid and practical IC test kit (ADTEC, Oita, Japan) for the detection of *P. gingivalis*, a key periodontopathic bacterium. Although the IC test yields results within 10 min, visual interpretation based on color development is inherently quantitative. Therefore, we additionally developed a dedicated reader system that enables quantitative measurement of the signal obtained from the IC kit. This integrated IC kit–reader system is expected to facilitate accurate, rapid, and reproducible chairside detection of *P. gingivalis*, thereby enhancing the diagnosis and monitoring of periodontitis and supporting broader investigations into its association with systemic diseases.

## 2. Materials and Methods

### 2.1. Preparation of Bacterial Suspensions for Evaluation

*P. gingivalis* (JCM12257 and JCM19600), obtained from the RIKEN BioResource Research Center Microbe Division (Ibaraki, Japan), was inoculated into bouillon medium and incubated anaerobically at 37 °C for 24 h using AnaeroPak^®^ Kenki (Mitsubishi Gas Chemical Company, Tokyo, Japan) to obtain an initial bacterial suspension (1.0 × 10^7^ CFU/mL). The bouillon medium was prepared by dissolving 3.0 g of trypticase soy broth, 0.5 g of yeast extract, 0.05 g of L-cysteine hydrochloride, 0.1 mL of hemin solution, and 0.02 mL of vitamin K_1_ solution in 100 mL of distilled water, followed by sterilization via autoclaving at 121 °C for 15 min. The hemin solution was prepared by dissolving 0.005 g of hemin and 0.0174 g of potassium hydrogen phosphate in 1 mL of distilled water. After anaerobic incubation, the bouillon culture was serially diluted 10-fold with sterile saline and plated onto Center for Disease Control (CDC) 5% sheep blood agar for anaerobes (Becton Dickinson and Company, Franklin Lakes, NJ, USA). The plates were incubated anaerobically at 37 °C for 48 h, and colony-forming units were counted. Based on these counts, the bacterial suspension was adjusted to a final concentration of 2.6 × 10^8^ colony-forming unit (CFU)/mL and stored at −80 °C until use).

### 2.2. Preparation of the IC Kit

**Figure 1:**
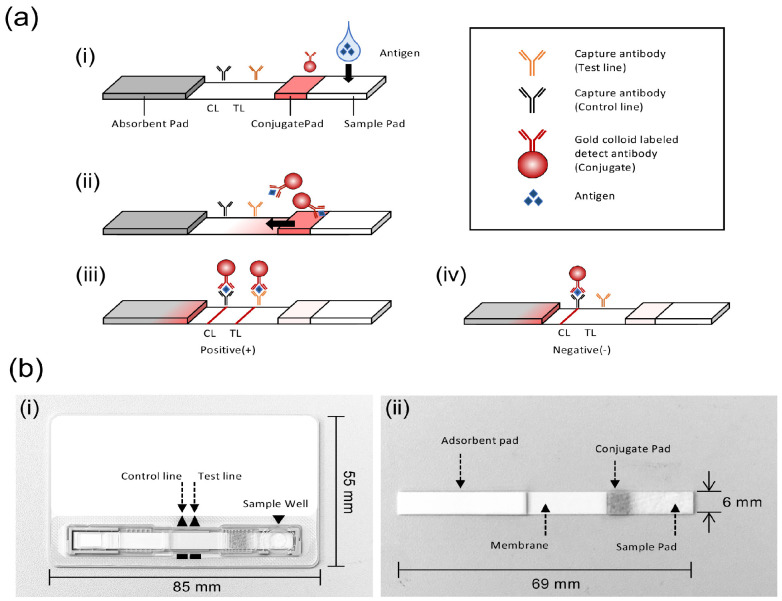
(**a**) Principle of the immunochromatography assay (**i**) A sample containing the target antigen is applied to the sample pad. (**ii**) Gold colloid–labeled antibodies migrate along the membrane and bind to the antigen. (**iii**) Positive result: Visible red lines appear at both the test (TL) and control (CL) lines. (**iv**) Negative result: A red line appears only at the control line (CL). (**b**) Developed immunochromatography test kit. (**i**) test cassette, (**ii**) test strip.

The IC kits consisted of a test cassette and specimen extraction solution. The test strips housed within the cassette were assembled using a nitrocellulose membrane, a sample pad (ADTEC), an absorbent pad (ADTEC), and a conjugate pad (ADTEC). Notably, these sample pads and the gold colloids were developed in-house specifically for this kit. The assembled test strips measured 68 mm in length and 6 mm in width. The membrane, composed of a microporous material with capillary properties, enabled lateral flow of the sample upon application. Specific monoclonal antibodies against *P. gingivalis* were obtained and purified from hybridoma cell lines using Protein G affinity chromatography (capture antibody; 1.45 mg/mL and detect antibody; 1.14 mg/mL). Sodium dodecyl sulfate–polyacrylamide gel electrophoresis (SDS-PAGE) confirmed that the purity of the antibodies was sufficient for use in the IC kit (Supplementary Figure S1). The membrane was coated with a capture antibody specific for *P. gingivalis* (ADTEC). A gold colloid-labeled antibody for *P. gingivalis* (ADTEC), was immobilized onto glass fiber (ADTEC), dried, and used as the conjugate pad. The test strips were then assembled into plastic cassette and used for subsequent experiments. The specimen extraction solution was prepared by dissolving 1% Triton X-100 (Sigma-Aldrich, St. Louis, MO, USA) in 10% phosphate-buffered saline (PBS) and clarifying it through a 0.45 μm filter (Sartorius AG, Göttingen, Germany) to remove fine particles and insoluble aggregates (Figure 1).

### 2.3. Protocol for Detection of P. gingivalis Using the IC Kit

For detection, 100 µL of the bacterial suspensions for evaluation, which were prepared at various concentrations with the sample extract (1% Triton X-100, 10% PBS), was applied to the sample drop region of the test card. After incubation for 10 min at room temperature, the presence of red lines in the test [Test] and control [Control] regions was visually examined. According to the acceptance criteria, a positive result was defined as the presence of a visible red line in the Test region and was considered indicative of reactivity. The lowest bacterial concentration at which a visible test line was detected among the serially diluted samples was identified and defined as the minimum detection limit for this strain using the IC kit. In establishing the protocol, we optimized the combination and concentrations of the capture and gold colloid-labeled detection antibodies, the blocking and buffer compositions, and the selection of the nitrocellulose membrane to maximize the signal-to-noise ratio, minimize background noise, and achieve the optimal maximum detection limit.

### 2.4. Cross-Reactivity Test

Bacterial strains were sourced from the ATCC (Manassas, VA, USA), the NITE Biological Resource Center (NBRC, Chiba, Japan), and the Japan Collection of Microorganisms (JCM, RIKEN BioResource Research Center). The viral strains used in this study were obtained exclusively from ATCC. Culture media containing each test microorganism were diluted with the specimen extraction solution. The concentration of each microorganism added to the samples was set at the maximum achievable level for each material. Each prepared sample (100 μL) was applied to the sample application port of the test cassette. After incubation for 10 min at room temperature, the presence of red lines in the test (Test) and control (Control) regions was visually examined. A negative result, defined as the absence of a visible line in the test region, was considered to meet the acceptance criterion. For microorganisms that yielded a positive (non-compliant) result at the initial test concentration, serial dilutions were performed using the specimen extraction solution to determine the highest concentration at which no test line was observed. This concentration was defined as the limit concentration showing no cross-reactivity.

### 2.5. Reaction Tests in the Presence of Coexisting Substances

The potential effects of substances that may be present in the oral cavity on the reaction system of the IC kit (measurement process and test results) were evaluated. Test samples consisted of weak-positive controls (3.85 × 10^5^ CFU/mL) and negative controls prepared by adding whole blood collected from healthy individuals (Sysmex, Kobe, Japan) and pharmaceutical agents listed in Table 1 at the concentrations specified in the test result sheet. All products were commercially available in Japan at the time of testing. The control samples comprised weak-positive controls and negative controls without the addition of any coexisting substances. The concentrations of pharmaceuticals and other coexisting substances added to the samples were set at the maximum achievable levels. Acceptance criteria were defined as follows: (i) test results identical to those obtained for the corresponding control samples without coexisting substances (i.e., positive results for weak-positive controls and negative results for negative controls), and (ii) no invalid test outcomes, such as disappearance of the control line. If a coexisting substance produced a non-compliant result at the test concentration, the sample was further diluted to determine the concentration at which compliance was achieved. The highest concentration at which the acceptance criteria were met was defined as the concentration of the coexisting substance that did not interfere with the reaction system of the IC kit.

### 2.6. Development of the Measurement Reader

**Figure 2:**
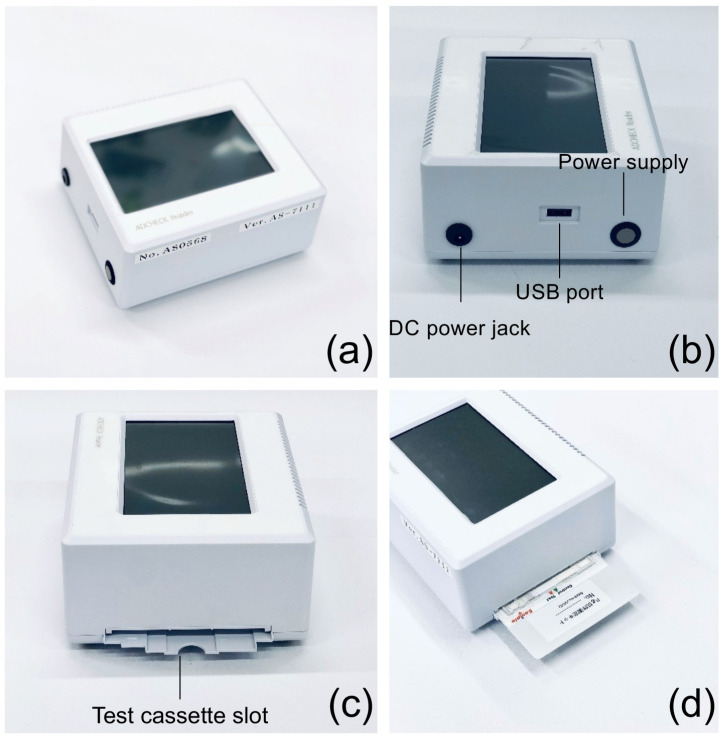
Overview and operational interface of the developed measurement reader. (**a**) External view of the measurement reader. (**b**) Side panel showing the power button, DC input jack for the AC adapter, and USB interface port. (**c**) Close-up view of the test card insertion slot. (**d**) Measurement configuration after insertion of the test card.

**Figure 3:**
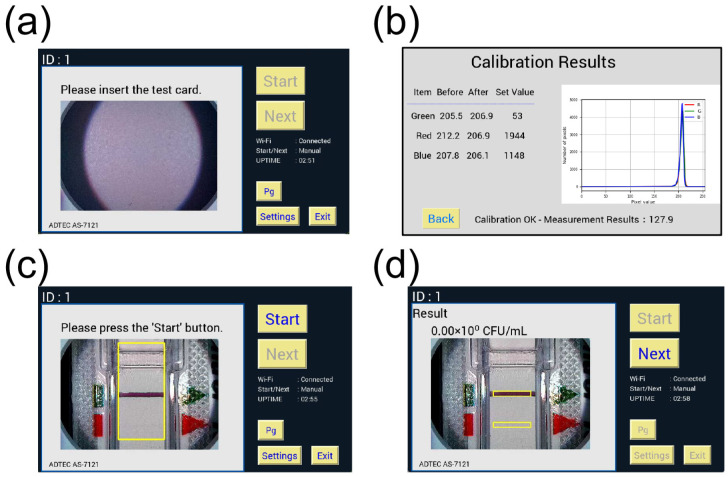
Measurement procedure for the immunochromatography reader. (**a**) Power on the device. (**b**) Perform baseline system calibration via the settings menu to ensure optical accuracy. (**c**) Insert the processed test card into the dedicated slot for measurement. (**d**) The calculated results are displayed on the screen and stored on a USB memory device.

The measurement reader was constructed using a Raspberry Pi 4 single-board computer (Raspberry Pi Foundation, Cambridge, UK) equipped with a 5-inch liquid crystal display (LCD) monitor, and the housing was fabricated via injection molding (Figure 2a,b). The measurement software (ADTEC) was developed using the Python 3.9.2 programming language. Measurement was initiated by inserting the cassette into the reader and pressing the judgment button (Figure 2c,d). The results were automatically stored on a universal serial bus (USB) memory device. For quantitative analysis, the a* parameter of the CIE L*a*b* color space was used as an index of chromatic intensity (Figure 3).

To verify the performance of the developed measurement reader, intradevice precision (repeatability) was evaluated by repeated measurements using a single reader. A pre-reacted positive test card (*P. gingivalis* ATCC33277 2.8 × 10^6^ CFU/mL), a weak positive test card (*P. gingivalis* ATCC33277 2.8 × 10^5^ CFU/mL), and a negative test card were used as test samples. Each card was measured 25 consecutive times using the same reader. The a* values were extracted from the acquired image data for quantitative analysis. The coefficients of variation (CVs) were calculated using the formula described below to assess measurement variability within the device.

CV (%) = standard deviation/average × 100

To assess interdevice precision, a comparison study was conducted using three independent measurement readers. *Porphyromonas gingivalis* strain JCM12257 culture suspensions were serially diluted with the sample extraction solution to prepare test samples ranging from 0 to 2.6 × 10^7^ CFU/mL. Each dilution was applied to a test card. After the specified reaction time, the a* value of each test card was measured using the three readers. The CV was calculated to evaluate interdevice measurement variability.

## 3. Results

### 3.1. Detection Performance of the IC Kits for Different Fimbrial Types of P. gingivalis

**Figure 4:**
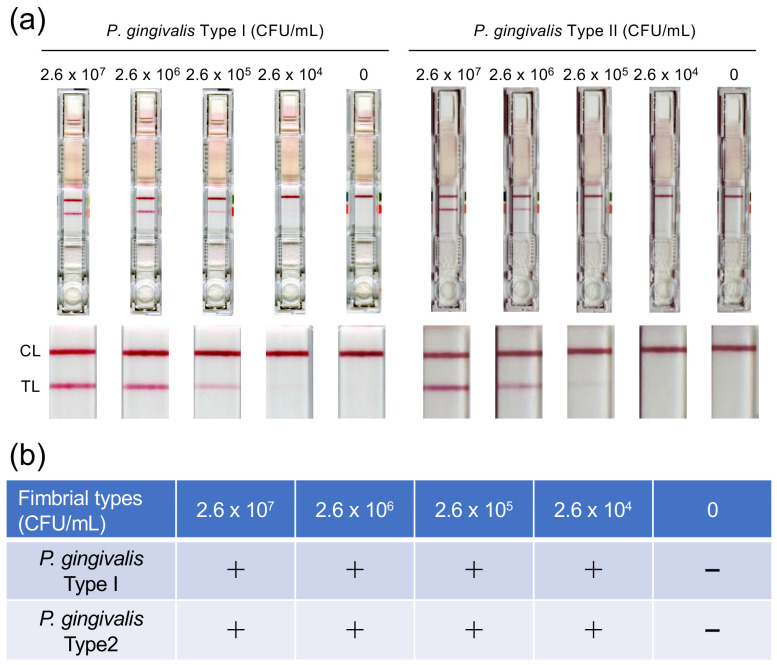
Evaluation of detection sensitivity for different fimbrial types of *Porphyromonas gingivalis*. (**a**) Representative images of the test strips showing the intensity of the test line at each bacterial concentration. Enlarged images of the test area are shown at the bottom. CL: control line; TL: test line (**b**) Table summarizing positive (+) and negative (−) results across serial dilutions.

The performance of the IC kit was evaluated by analyzing test results at different bacterial concentrations. Result interpretation was defined according to the kit’s instructions: the appearance of a red line in the [Test] zone was interpreted as positive (+), whereas the absence of a red line was interpreted as negative (−). Results were to be considered invalid when no red line appeared in the [Control] zone; however, no invalid results were obtained during this evaluation. The negative control consistently yielded negative results, and no false-positive reactions attributable to non-specific binding were observed. The detection capability of the IC kit was evaluated using the *P. gingivalis* strains representing different fimbriae types: Type I (JCM12257) and Type II fimbriae (JCM19600). The IC kit produced a clearly visible test line at bacterial concentrations as low as 2.6 × 10^4^ CFU/mL for both strains (Figure 4).

### 3.2. Cross-Reactivity Test of the IC Kit

To evaluate the analytical specificity of the IC kit, cross-reactivity testing was performed using representative bacteria, fungi (Table 2), and viruses (Table 3) that are expected to be present in oral specimens. At the concentrations tested, none of the evaluated microorganisms yielded positive test results, indicating the absence of detectable cross-reactivity under the conditions of this study.

### 3.3. Performance of the IC Kit in the Presence of Coexisting Substances

We evaluated the effect of substances that may be present in the oral cavity in daily life settings on the measurement process and analytical performance of the IC kit. Samples containing 0.5% and 1% blood met the compliance criteria; however, testing with a sample containing 2% blood produced a positive result in the negative control. Under other coexisting conditions involving pharmaceuticals and various substances, all samples met the compliance criteria for the kit at the tested concentrations (Table 4).

### 3.4. Validation of Reader Accuracy and System Precision

**Figure 5:**
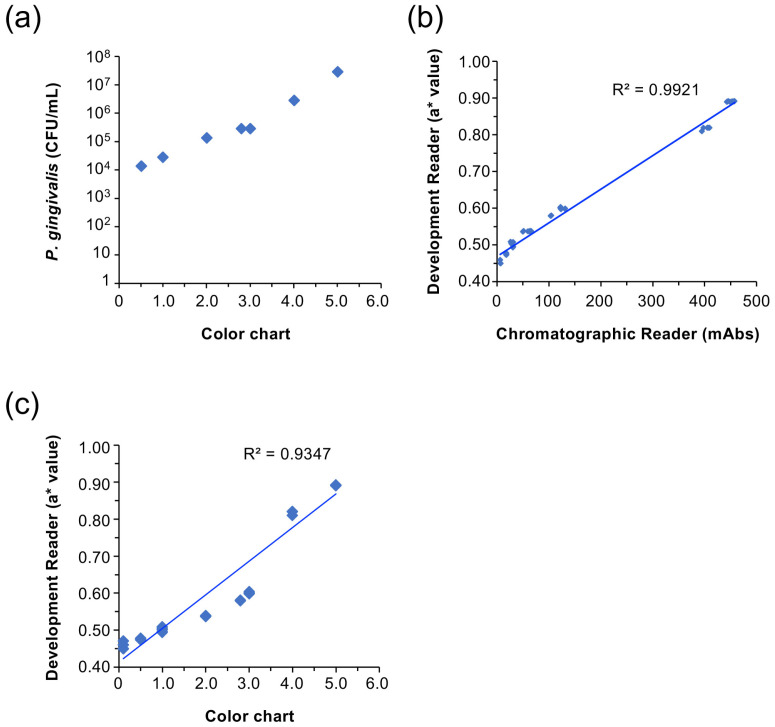
Performance evaluation and correlation analysis of the developed measurement reader. (**a**) Visual evaluation using a standard bacterial strain (*Porphyromonas gingivalis* ATCC 33277) (**b**) Correlation analysis between the developed reader and a reference immunochromatographic reader. (**c**) Correlation analysis between the reader output and a standard visual color chart.

The reliability of the developed measurement reader was validated through correlation analyses using a standard bacterial strain (Figure 5a). As shown in Figure 5b, the measurement values (a* values) obtained using the developed reader showed a strong linear correlation (*R*^2^ = 0.9921) with the absorbance data measured using a high-precision reference immunochromatographic reader (C10066; Hamamatsu Photonics, Hamamatsu City, Japan). In addition, the numerical outputs from the developed reader exhibited a strong positive correlation (*R*^2^ = 0.9347) with visual scores determined using a standard color chart, which is commonly applied in clinical assessments (Figure 5c).

### 3.5. Evaluation of Intra-Device Reproducibility and Inter-Device Measurement Error of the Measurement Reader

To evaluate the intra-device reproducibility of the measurement reader, 25 consecutive measurements were performed using negative and positive controls. The results indicated narrow ranges between maximum and minimum values and low standard deviations in both groups, indicating high measurement stability. The CVs, used as indicators of reproducibility, were <1.0% for positive (0.08%), weak-positive (0.52%), and negative (0.54%) controls (Table 5). Comparative testing across three separate reader units demonstrated excellent reproducibility, with CV <0.6% for all the tested concentrations (Table 6).

## 4. Discussion

Detecting *P. gingivalis*, one of the most important periodontal pathogens, is crucial for effective management of periodontitis. Several methods are used to detect periodontal pathogens. Real-time PCR allows for the quantification of trace bacterial amounts through DNA amplification. However, the equipment required for PCR is bulky, and rapid detection is difficult, making it unsuitable for chairside testing. Furthermore, the high cost of analysis remains a significant challenge for routine PCR-based diagnosis.

IC offers the advantages of shorter measurement times and rapid testing compared with other diagnostic methods [20,21,22]. Performance evaluation of the kit developed in this study revealed a dose-dependent increase in the intensity of IC coloration in samples containing *P. gingivalis*. These findings indicate that the gold colloid-labeled antibodies in the IC kit formed immune complexes with *P. gingivalis* in the test solution, which were subsequently captured by antibodies immobilized on the membrane as the sample migrated along the strip. In contrast, no test lines were observed in specimens lacking *P. gingivalis*, supporting the high specificity of the antigen–antibody reaction employed in the IC kit.

Several *P. gingivalis* strains are known to exist, exhibiting a high degree of genetic diversity [23]. The long fimbriae of *P. gingivalis* are classified into six types (I–V and Ib) based on nucleotide sequence variations in the fimbrial protein A (*fimA*) gene [24]. PCR analysis of subgingival plaque samples showed that *fimA* type I is the most frequently detected in healthy adults, whereas type II is the most prevalent in patients with periodontitis, followed by type IV [25]. The strain JCM19600 (*fimA* type II) was isolated from patients with severe periodontitis and has been reported to exhibit high pathogenicity [26]. In the present study, we could detect JCM19600 as well as ATCC33277 (*fimA* type I) using the developed IC kit, indicating the applicability of this kit for the detection of multiple *P. gingivalis* strains. In contrast, strain W83 (*fimA* type IV) has been reported to strongly influence symbiotic bacterial species [27] and induce alveolar bone loss in a periodontitis mouse model [28]. We are currently evaluating the reactivity of the IC kit against strain W83 and other *fimA* types. Further validation across additional clinically relevant *fimA* genotypes will help clarify the broad applicability of the IC kit.

Given the o complex microbiota of the oral cavity [29] and its role as a gateway for the entry of exogenous microorganisms, high specificity is an essential requirement for IC kits designed for use with oral samples. The IC kit developed in this study yielded positive results for *P. gingivalis* and negative results for other tested pathogens, indicating a high degree of specificity for the detection of *P. gingivalis*.

To further evaluate the reliability of the developed kit, we investigated its detection performance in the presence of common oral care products, such as toothpastes and mouthwashes. These agents at concentrations of 10 mg/mL did not affect the ability of the IC kit to detect *P. gingivalis*. These findings suggest that the IC kit may be applicable to oral specimens, which often contain a variety of exogenous substances. However, accurate bacterial detection was not possible in samples containing more than 2% blood. Saliva from patients with periodontitis has been reported to contain hemoglobin equivalent to approximately 0.53 μL of blood (0.58 μg/mL) [30]. In addition, hemoglobin levels in GCF are significantly elevated (1116.6 nM) compared with those in healthy individuals (46.6 nM) [31]. Therefore, further improvements in detection sensitivity may be required for this IC kit to achieve clinically meaningful performance as an in vitro diagnostic tool for periodontitis. We are currently conducting performance evaluations using clinical specimens, including GCF, dental plaque, and saliva.

The a* values obtained using the measurement reader developed in this study showed a strong correlation with the absorbance values measured using a reference IC reader (*R*^2^ = 0.9921). The measurement reader employs a camera-based image analysis technology, enabling automatic detection of the colored test lines without the need for specialized mechanical driving components. As a result, the device can be designed as a compact, palm-sized reader.

The lower sensitivity compared with that of PCR is often cited as a limitation of IC. The fabricated IC kit was capable of detecting *P. gingivalis* at concentrations as low as 2.6 × 10^4^ CFU/mL; however, the test line was visualized as an extremely faint line at this concentration, and some cases were interpreted as negative. As shown in Figure 2, signals below 5.0 mAbs were also difficult to detect visually using the IC reader, suggesting that the practical lower limit of detection by visual inspection was approximately 2.6 × 10^4^ CFU/mL. For the developed measurement reader, the difference in a* values between 0 and 2.6 × 10^4^ CFU/mL was minor (0.27) and was considered to be equivalent to the lower detection limit of visual inspection. Previous studies using quantitative PCR have shown that the mean number of *P. gingivalis* in subgingival samples from patients with periodontitis was 4.42 × 10^6^ CFU/mL, which was significantly higher than that in healthy subjects (1.57 × 10^4^ CFU/mL) [32]. Another study also revealed that the concentration of *P. gingivalis* in subgingival plaque or saliva strongly correlates with active periodontal destruction when it reaches a threshold of 10^4^ to 10^5^ cells/mL [33]. Based on these findings, the developed IC kit is expected to exhibit sufficient sensitivity for the detection of *P. gingivalis* in patients with periodontal disease. In fact, in a study using a prototype reader to analyze subgingival plaque from patients with periodontitis, the results of *P. gingivalis* detection using this IC kit showed significant positive correlations with both real-time PCR results and periodontal tissue parameters [34]. Furthermore, the developed reader-based measurement method exhibited high reproducibility, enabling stable quantitative measurements across different reader units.

In addition to *P. gingivalis*, the other Red Complex species, *T. denticola* and *T. forsythia*, are also known to play a pivotal role in the pathogenesis of periodontitis [35]. In a systematic review, the frequency of detection of Red Complex strains in patients with periodontitis has been reported to be >60% in most studies, regardless of the type of specimen, such as saliva or subgingival plaque [36]. We previously developed a test kit for quantifying the activity of N-benzoyl-DL-arginine peptidase (trypsin-like peptidase) produced by periodontopathogenic bacteria and demonstrated its utility as a simple and rapid method for detecting severe periodontitis [37]. We also reported that elevated trypsin-like peptidase activity detected in tongue swabs was associated with decreased kidney function [38]. However, because *T. forsythia* and *T. denticola* also produce the same peptidase, the specific detection of *P. gingivalis* remains impossible using this kit. In combination with the IC kit developed in this study, the approach has the potential to enable a simpler and more accurate periodontal disease diagnostic method for periodontal disease. In addition to the pathology of periodontitis, it may also contribute to the development of examination methods that enhance the understanding of the relationship between periodontitis and lifestyle-related diseases.

## 5. Conclusions

The IC kit developed in this study demonstrated the capability for sensitive and quantitative detection of *P. gingivalis* within approximately 10 min. The detection limit of this kit was 2.6 × 10^4^ CFU/mL, with no cross-reactivity with oral commensal bacteria and was no interference from co-existing substances. Furthermore, the dedicated reader developed for the quantitative evaluation of immunochromatographic detection bands demonstrated a low CV in both intra- and inter-device reproducibility, indicating high reliability for quantitative assessment. Future studies using clinical specimens are expected to lead to their practical application as a chairside diagnostic tool for periodontal disease. In addition, we will continue clinical performance testing with a view to obtaining regulatory approval as an in vitro diagnostic (IVD) device, with the ultimate goal of establishing a comprehensive, next-generation periodontal disease diagnostic system through the combination with enzyme activity kits and other diagnostic tools.

## Figures and Tables

**Table 1:** Coexisting substances used in the interference study.

Category	Coexisting Substance (Commercial Product)	Manufacturer
Mouthwash 1	Mondahmin PX4	Earth Corporation (Tokyo, Japan)
Mouthwash 2	Listerine CM	Kenvue (Summit, NJ, USA)
Mouthwash 3	Lacleche^®^	JEX Co., Ltd. (Osaka, Japan)
Liquid dentifrice	G·U·M WN	Sunstar Inc. (Osaka, Japan)
Paste dentifrice 1	Deep Clean SB	Kao Corporation (Tokyo, Japan)
Paste dentifrice 2	Yakuyō Lacalut New 5	SSP Co., Ltd. (Tokyo, Japan)
Oral antiseptic	Isodine Gargle C	Meiji Co., Ltd. (Tokyo, Japan)
Throat lozenge/candy 1	Colgen Kowa Troche	Kowa Company, Ltd. (Aichi, Japan)
Throat lozenge/candy 2	Gotosan Throat Candy	Irie Seika Co., Ltd. (Fukuoka, Japan)
Throat lozenge/candy 3	Propolis & Manuka Honey Throat Candy	Asada Ame Co., Ltd. (Tokyo, Japan)

**Table 2:** Results of cross-reactivity test for bacteria and fungi.

Bacteria	Strain No.	Concentration (CFU/mL)	Detection
*Bordetella pertussis*	NBRC107857	1.69 × 10^8^	−
*Candida albicans*	NBRC1385	3.25 × 10^8^	−
*Citrobacter freundii*	JCM1657	2.24 × 10^9^	−
*Corynebacterium diphtheriae*	JCM1310	5.25 × 10^6^	−
*Enterococcus durans*	NBRC100479 (Lancefield’s Group D)	3.75 × 10^8^	−
*Enterococcus faecalis*	NBRC100480 (Lancefield’s Group D)	6.00 × 10^8^	−
*Escherichia coli*	ATCC11775 (JCM1649)	2.40 × 10^9^	−
*Haemophilus influenzae*	ATCC9006	1.56 × 10^8^	−
*Klebsiella pneumoniae*	ATCC13883 (JCM1662)	1.76 × 10^8^	−
*Listeria monocytogenes*	JCM7671	1.07 × 10^9^	−
*Moraxella catarrhalis*	ATCC8176	3.70 × 10^9^	−
*Mycoplasma orale*	NBRC14477	2.48 × 10^7^	−
*Mycoplasma pneumoniae*	NBRC14401	1.81 × 10^7^	−
*Mycoplasma salivarium*	NBRC14478	1.02 × 10^6^	−
*Mycoplasma hominis*	NBRC14850	1.34 × 10^5^	−
*Neisseria gonorrhoeae*	ATCC19424	1.02 × 10^9^	−
*Neisseria meningitidis*	ATCC13077	4.20 × 10^9^	−
*Neisseria sicca*	ATCC9913	5.20 × 10^9^	−
*Neisseria subflava*	ATCC19243	4.10 × 10^9^	−
*Prevotella intermedia*	JCM11150	1.19 × 10^9^	−
*Proteus vulgaris*	NBRC3045	4.25 × 10^9^	−
*Pseudomonas aeruginosa*	NBRC12689	3.85 × 10^9^	−
*Salmonella enteritidis*	IFO3313	1.15 × 10^9^	−
*Salmonella typhimurium*	IFO13245	8.20 × 10^9^	−
*Serratia marcescens*	NBRC3046	1.04 × 10^9^	−
*Staphylococcus aureus*	NBRC102135	3.17 × 10^7^	−
*Staphylococcus epidermidis*	NBRC100911	1.38 × 10^8^	−
*Streptococcus agalactiae*	JCM5671 (Lancefield’s Group B)	1.79 × 10^8^	−
*Streptococcus anginosus*	JCM12993 (Lancefield’s Group G)	2.08 × 10^8^	−
*Streptococcus constellatus* subsp. *constellatus*	JCM12994	5.05 × 10^8^	−
*Streptococcus dysgalactiae* subsp. *dysgalactiae*	JCM5673 (Lancefield’s Group C)	4.04 × 10^8^	−
*Streptococcus oralis*	JCM12997	8.60 × 10^7^	−
*Streptococcus mitis*	JCM12971	1.75 × 10^8^	−
*Streptococcus mutans*	NBRC13955	4.25 × 10^8^	−
*Streptococcus mutans*	JCM5705	1.11 × 10^9^	−
*Streptococcus oralis* subsp. *oralis*	JCM12997	2.85 × 10^8^	−
*Streptococcus pneumoniae*	NBRC102642	6.45 × 10^9^	−
*Streptococcus pyogenes*	JCM5674 (Lancefield’s Group A) T1	1.07 × 10^8^	−
*Streptococcus pyogenes*	ATCC12353 (T12)	8.95 × 10^8^	−
*Streptococcus pyogenes*	ATCC12962 (T28)	9.65 × 10^8^	−
*Streptococcus pyogenes*	ATCC12204 (T25)	1.11 × 10^9^	−
*Streptococcus pyogenes*	BAA-1066 (M4)	8.35 × 10^8^	−
*Streptococcus salivarius*	JCM5707	4.50 × 10^8^	−
*Streptococcus sanguinis*	JCM5708	2.85 × 10^7^	−
*Streptococcus sobrinus*	JCM33478	5.60 × 10^8^	−
*Tannerella forsythia*	JCM10827	8.05 × 10^8^	−

**Table 3:** Results of cross-reactivity test for viruses.

Virus	Strain No.	Concentration (TCID_50_/mL)	Detection
*Adenovirus 3*	ATCC VR-3	1.0 × 10^6^	−
*Adenovirus 6*	ATCC VR-6	2.5 × 10^6^	−
*Adenovirus 7*	ATCC VR-7	7.5 × 10^4^	−
*Coxsackievirus B3*	ATCC VR-30	7.5 × 10^6^	−
*Echovirus 6*	ATCC VR-240	7.5 × 10^6^	−
*Echovirus 16*	ATCC VR-46	7.5 × 10^5^	−
*Echovirus 32*	ATCC VR-324	7.5 × 10^5^	−
*Herpes Simplex Virus Type* *1*	ATCC VR-735	5.0 × 10^3.25^	−
*Herpes Simplex Virus Type* *2*	ATCC VR-734	5.0 × 10^4.75^	−
*Parainfluenza Virus Type 2*	ATCC VR-92	7.5 × 10^5^	−
*Parainfluenza Virus Type 3*	ATCC VR-93	7.5 × 10^6^	−

**Table 4:** Effect of coexisting substrates on test results.

Coexisting Substances	Concentration	Sample	Detection	Acceptance
Blood	2%	PgWPC	+	Not accept
		NC	+	
	1%	PgWPC	+	Accept
		NC	−	
	0.5%	PgWPC	+	Accept
		NC	−	
Mouthwash 1	10%	PgWPC	+	Accept
		NC	−	
Mouthwash 2	10%	PgWPC	+	Accept
		NC	−	
Mouthwash 3	10%	PgWPC	+	Accept
		NC	−	
Liquid dentifrice	10%	PgWPC	+	Accept
		NC	−	
Paste dentifrice 1	10 mg/mL	PgWPC	+	Accept
		NC	−	
Paste dentifrice 2	10 mg/mL	PgWPC	+	Accept
		NC	−	
Oral antiseptic	10 mg/mL	PgWPC	+	Accept
		NC	−	
Throat lozenge/candy 1	10 mg/mL	PgWPC	+	Accept
		NC	−	
Throat lozenge/candy 2	10 mg/mL	PgWPC	+	Accept
		NC	−	
Throat lozenge/candy 3	10 mg/mL	PgWPC	+	Accept
		NC	−	

PgWPC; weak-positive controls (*P. gingivalis* 3.85 × 10^5^ CFU/mL). NC; negative control.

**Table 5:** Summary of intradevice reproducibility (*n* = 25).

Description	Mean	SD	CV
Negative	0.456	0.0025	0.54%
Weak Positive	0.601	0.0031	0.52%
Positive	0.681	0.0005	0.08%

**Table 6:** Summary of interdevice measurement.

Description		Device 1	Device 2	Device 3
Negative	Mean	0.4640	0.4556	0.4471
	SD	0.0010	0.0025	0.0019
	CV	0.219%	0.538%	0.427%
Weak-positive	Mean	0.6167	0.6006	0.6049
	SD	0.0011	0.0031	0.0008
	CV	0.177%	0.517%	0.130%
Positive	Mean	0.6682	0.6815	0.6676
	SD	0.0026	0.0005	0.0009
	CV	0.383%	0.077%	0.128%

## Data Availability

The data presented in this article are available upon request from the corresponding author.

## Supplementary Materials

The following supporting information can be downloaded at: https://www.mdpi.com/article/10.3390/dj14070422/s1. Figure S1: Representative photo of results for SDS-PAGE. (M; molecular weight marker, 1; ascites, 2; throughout fraction, 3, purified fraction).

## Abbreviations

The following abbreviations are used in this manuscript:

LPS	Lipopolysaccharide
GCF	Gingival crevicular fluid
PCR	Polymerase chain reaction
IC	Immunochromatography
CDC	Center for Disease Control
CFU	Colony-forming units
SDS-PAGE	Sodium dodecyl sulfate–polyacrylamide gel electrophoresis
PBS	Phosphate-buffered saline
ATCC	American Type Culture Collection
NBRC	NITE Biological Resource Center
JCM	Japan Collection of Microorganisms
LCD	liquid crystal display
USB	Universal serial bus
FimA	Fimbrial protein A
