# Supplementary material for: Development of a Quantitative Detection System for Porphyromonas gingivalis Based on Immunochromatography
Source: Dent J (Basel). 2026 Jul 9;14(7):422. doi: 10.3390/dj14070422 (PMC13407973; doi:10.3390/dj14070422)
Supplement: Supplementary file 1 [file dentistry-14-00422-s001.zip › dentistry-4258411-supplementary.pdf]

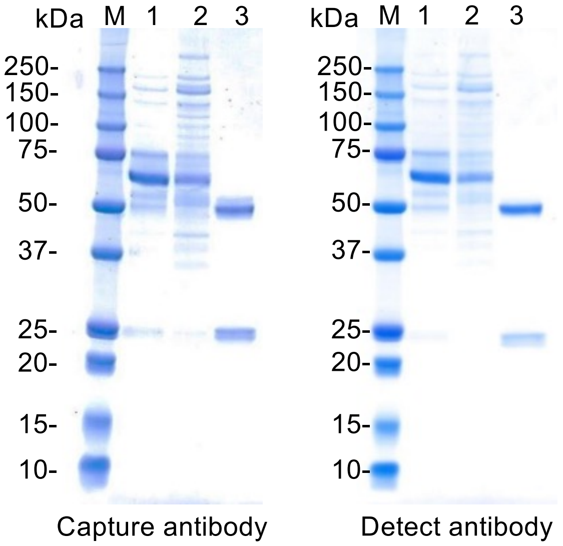

Supplemental Figure S1 Representative photo of results for SDS-PAGE. (M; molecular weight marker, 1; ascites, 2; throughout fraction, 3, purified fraction.
